# The Etiology of Parkinson’s Disease: New Perspectives from Gene-Environment Interactions

**DOI:** 10.3233/JPD-230250

**Published:** 2023-12-19

**Authors:** Jolien S. Bogers, Bastiaan R. Bloem, Jonas M. Den Heijer

**Affiliations:** a Radboud University Nijmegen Medical Center, Donders Institute for Brain, Cognition and Behavior, Department of Neurology, Nijmegen, the Netherlands; b Amsterdam University Medical Center, Department of Neurology, Amsterdam, the Netherlands

**Keywords:** Parkinson’s disease, etiology, genetics, environment, pesticides, MPTP

## Abstract

Parkinson’s disease is now the most rapidly growing neurodegenerative disease worldwide. It is therefore critical to identify which factors, and to what extent, contribute to the multifactorial etiology of Parkinson’s disease. Here, we address two interesting elements from the perspective of genetics, namely (a) the estimated age of several genetic risk factors related to Parkinson’s disease; and (b) the relative contribution of genetics to the etiology of Parkinson’s disease, as derived from twin studies. Based on these two perspectives, we argue that most genetic risk factors are by themselves insufficient to explain the majority of Parkinson’s disease, and that environmental factors are required for these genetic factors to become pathophysiologically relevant.

## INTRODUCTION

Prior to 1817, when James Parkinson was the first to describe the characteristics of what is now known as Parkinson’s disease (PD) [1], this neurological disease was likely rare [2]. However, in the ensuing two centuries, the incidence and prevalence of PD have risen sharply worldwide. This growth appears to be accelerating, with recent data suggesting that between 1990 and 2015, the number of affected people rose from 2.6 to 6.3 million. It is expected that by the year 2040, this number may have risen further to some 13 million [3].

There are various factors that might explain this rapid growth. Aging of our population certainly contributes— it is well established that age is a major risk factor for PD [4]. However, the growth of PD persists even after correction for this ageing effect, with an age-corrected increase in prevalence of 22% [5]. This makes PD the fastest growing neurological disease in the world [3].

Importantly, apart from aging, other causative factors appear to be at play as well. First, there is increasing attention for the role played by exposure to environmental toxins such as pesticides, air pollution or trichloroethylene [6, 7]. Despite the efforts that have been made to control some risk factors (e.g., banning certain pesticides), PD is still on the rise. There may be several explanations for this: (1) the effect of such protective measures will not be observed directly, due to the long prodromal period of PD; (2) most pesticides are environmentally persistent, meaning that continued exposure takes place from residues in our environment; (3) many other neurotoxic pollutants still persist and could contribute to causing PD, either alone or because of co-exposure [8]— recent work points to the role of, e.g., air pollution [9, 10], and a particular concern in this regard is that none of the currently used pesticides can be regarded as safe from a PD perspective because current regulatory actions do not adequately assess the PD risks [11]; and (4) the aging of our population— age itself is unlikely to be a cause of PD per se, but the fact that people live longer allows them to exposed to pesticides and other environmental toxicants during a longer exposure period. Just how large this environmental contribution to PD is, and particularly what the relative contribution is of specific environmental toxins, remains difficult to estimate.

Second, there is also a genetic contribution to PD, including several autosomal dominant and recessive factors. The research topic of genetics in the field of PD is relatively young but is growing exponentially. The first genetic link with PD was discovered only quite recently, in 1997. Specifically, a mutation in the *SCNA*-gene, encoding alpha-synuclein, was identified in families with a high prevalence of PD [12]. Thereafter, with increasing accessibility to genetic analyses, there was hope for a ‘genetic revolution’ [13]. Especially after the introduction of genome-wide association studies (GWAS), many other genetic variants have been associated with PD as well [14]. While our knowledge of the genetic contribution to PD has indeed increased enormously, the actual effect sizes remain limited for the majority of people with PD, as reflected by the relatively low familial aggregation of PD and by heritability estimates of only 0.14–0.22 [15]. Most of the genetic variants are risk factors with a low penetrance [16], and only 3–5% of cases of PD are explained by a monogenic form [4]. So overall, genetic abnormalities appear to explain only a relatively small proportion of the overall Parkinson population in the world.

Importantly, it would be difficult to explain the worldwide growth of PD solely by a contribution by these genetic factors, unless new mutations would have arisen in recent years, or when the involved genes would somehow interact with the changes in our environment. In this Hypothesis, we address how the undeniable contribution of genetic factors to the etiology of PD can be reconciled with what appeared to be a newly discovered disorder some two centuries ago, as well as its rapid growth in recent decades. We do this by addressing two specific elements related to the genetics of PD: the estimated age of mutations in genes that nowadays can be found quite commonly in the general PD population; and the relative contribution of genetics to the overall PD etiology, as derived from twin studies, placed in perspective relative to other common disorders with a mixed etiology. Based on this two-pronged evaluation, we argue that environmental factors must account for the lion’s share in the multifactorial etiology of PD.

## THE ESTIMATED AGE OF COMMON GENETIC FACTORS

If the etiology of PD would be determined predominantly by genetic causes, then such genetic contributions should have been rare prior to the year 1817, when this highly visible disease was described for the first time as a neurological condition on the streets in London. But interestingly, at least some of the genetic causes appear to have a much older age. We will here use *GBA1* as an example because it was possible to estimate the age for this very common genetic mutation. Indeed, apart from the GWAS risk loci, a variant in the *GBA1* gene is the most common genetic risk factor known to date, which can be found in some 5-15% of people with PD, depending on which specific part of the population is tested [17]. It is possible to estimate the age of specific mutations known to be involved in PD based on the publicly accessible database Atlas of Variant Age (https://human.genome.dating) [18]. This database, which is based on 2,782 individuals, estimates the age of a genetic variant in population-scale sequencing data using a nonparametric approach, referred to as Genealogical Estimation of Variant Age (GEVA), which is extensively explained elsewhere [18]. Based on the variant frequency during whole genome sequencing [18], the age of many *GBA1* risk variants are estimated to be over 7000 years old (Table 1).

**Table 1:** An overview of genetic factors associated with PD

**Gene**	**Protein**	**Frequency in PD**	**Environmental interactions**	Pathogenic variants*	**Presumed founder (years)**
Autosomal dominant	
**SNCA**	Alfa-synuclein	< <1%	Paraquat	A30P	?
			Rotenone	E46K	?
				H50Q	?
				G51D	?
				A53T	?
**LRRK2**	Leucine-rich repeat kinase 2	∼3%	Rotenone	G2019S	?
			Trichloroethylene	I2020T
			Paraquat	R1441C	?
				R1441G	?
				R1441H	?
				N1437H	?
				Y1699C	?
**VPS35**	Vacuolar protein sorting-associated protein 35	<1%	Rotenone	D620N	?
**Autosomal recessive**	
**Parkin**	Parkin	∼1%	Paraquat,	R42P	7,715
			Rotenone	V65E	?
				K211N	?
				C212Y	?
				T415N	?
				C431F	?
				C441R	?
				T240R	?
				R275W	4,358
				G430D	?
**PINK1**	PTEN induced putative kinase 1	<1%	Rotenone,	G309D	?
			MPTP	Q456X	?
				L347P	?
				G411S	?
				W437X	?
				M261I	?
**DJ-1**	Protein deglycase DJ-1	< <1%	Rotenone	L166P	?
				A104T	?
**Risk factor**
**GBA1**	Glucocerebrosidase	5-15%	MPTP	E326K	12,525
			Rotenone	N370S	7,003
				L444P	15,010
				D140H	?
				T369M	?
**LRRK2**	Leucine-rich repeat kinase 2	5–10%	?	G2385R	17,943
**GWAS risk variant**	Intergenic variant (i.e., non-protein-coding)	∼50%	?	rs6658353	1,036,045
**Example unrelated to PD**
**LRRK2**	Leucine-rich repeat kinase 2 (benign)			M2397T	888,858

This table provides an overview of genetic factors associated with PD, including their environmental interactions and estimated age based on human genome dating database [18]. *A selection of the most common pathogenic variants. For most genes several to tens of other variants exist, but for *GBA1* over 400 pathogenic variants have been reported thus far [17].

If having such a long-existing mutation would by itself have been a sufficient reason to develop PD, then there should have been many more descriptions of people with PD long before 1817. Only sporadic descriptions of some symptoms of PD can be found in the ancient literature, which highly disproportionate to the frequency seen nowadays, and which cannot merely be explained by aging [19]. The risk of developing PD is definitely age-related, so the fact that people died in a much younger age in earlier times can certainly explain why this was a much rarer condition in the past. But the same time, it seems nearly impossible that this remarkable and highly characteristic clinical phenotype would have gone almost unnoticed and undocumented for millennia. One might argue that the presence of parkinsonian signs that would have occurred in the relatively small proportion of people that did grow old, even in those days, might have been dismissed as a part of regular ageing. But this cannot be said of those that develop young-onset PD (i.e., an age at onset below 40 to 50 years of age), which is all but rare these days and which, if genetic were the dominant cause of PD, should have been equally prevalent in the past. An example is *GBA1*-associated parkinsonism, which is typically associated with a relatively younger age at onset [4, 17]. For example, in a Dutch cohort of 312 persons with GBA-PD, the mean age at onset was 5 years younger compared to noncarriers, with a range that extended to individuals as young as just 25 years of age [17]. Other genetic causes of PD are also typically associated with the young age at onset [20]. Clear signs of parkinsonism in such younger individuals should have attracted the attention of the excellent clinicians (who were typically astute observers) that have been around since ancient Greek times, unless all other mutations in PD genes arose only after 1817. Since apparently this is not the case, other factors that arose later must have somehow interacted with the predisposing underlying genetic factors, thus ultimately leading to PD. And we suspect that these additional factors might well be found in the environment, as gene-environment interactions are well documented for many different PD genes, including *GBA1*.

There is some evidence to support this assumption. For example, it is known that *GBA1* variants confer an estimated overall 2- to 7-fold increased risk to develop PD [17]. This means that most people with such a risk factor gene variant will not develop PD, implying an important contribution of additional, and arguably relatively new, factors. Thus far, it remains unclear what exactly determines which carriers of a *GBA1* variant will and which will not develop PD. In animal models, carriers of *GBA1* mutations are particularly vulnerable following exposure to environmental factors, such as the potent nigrostriatal neurotoxin MPTP or the pesticide rotenone [22, 23]. Similar gene-environment interactions exist for all of the genes that have been associated with an increased risk of developing PD (Table 1). And interestingly, these environmental factors often include exposure to pesticides, which have been widely implemented as a risk factor for developing PD, and which were only introduced widely into our society after the second World War, to ascertain that we could feed a rapidly growing world population.

Other monogenic forms of PD are also often associated with a young age at onset (even < 21 years of age) [4]. We do not know the exact age of these other genetic forms of parkinsonism, because the GEVA approach described above become less reliable or impossible for rare variant estimates, due to its limited sample size. In line with this, none of the autosomal dominant PD mutations can be found in the database (Table 1), since these are thought to be rare, de novo variants. Therefore, the contribution of the environment in these cases remains uncertain. Interestingly, however, experimental work indicates that environmental factors may also play a role for these other clearly genetically determined cases [24]. If these other genetic causes where the sole contributor to PD, we should have seen young-onset PD prior to 1817, unless these mutations are all new. It remains remarkable, however, that over the past 25 years multiple autosomal dominant mutations have been identified (multiple variants in *VPS35*, *SNCA*, and *LRRK2*), while theoretically such de novo mutations also could have happened in the last 10,000 years. This suggests that for these mutations to become fully penetrant, some interaction with toxins in our environment remains likely necessary.

Variants that are robustly described as risk factors for PD (like *GBA1*, but also many GWAS hits) can be very common and are estimated to be very old (Table 1). For example, a variant of *LRRK2* (G2385 R) with a high prevalence and a low penetrance (i.e., a risk factor), gives a slightly lower risk to develop PD compared to *GBA1* and is estimated to be 17,943 years old. Moreover, benign variants of *LRRK2* are even much older (e.g., M2397T is estimated to be 888,858 years old). Genetic risk factors identified through GWAS have even lower risks and can be estimated to be over a million years old (e.g., SNP rs6658353 [14]). The function of many of these (intronic) SNPs is still unclear, but subtle influences on metabolism seem a plausible hypothesis (for example, involvement in the processing and clearance of environmental toxins; note that cytochrome P450 variants have been associated with PD as well [25]). These examples underline that old mutations cannot have had a large effect on developing PD. Old mutations with a large effect have to contribute to a disease which is itself also old and widespread, such as breast cancer. Indeed, variants of *BRCA1* and *BRCA2* are estimated to be several millennia old [26].

## A NEW LOOK AT TWIN STUDIES

**Fig. 1:**
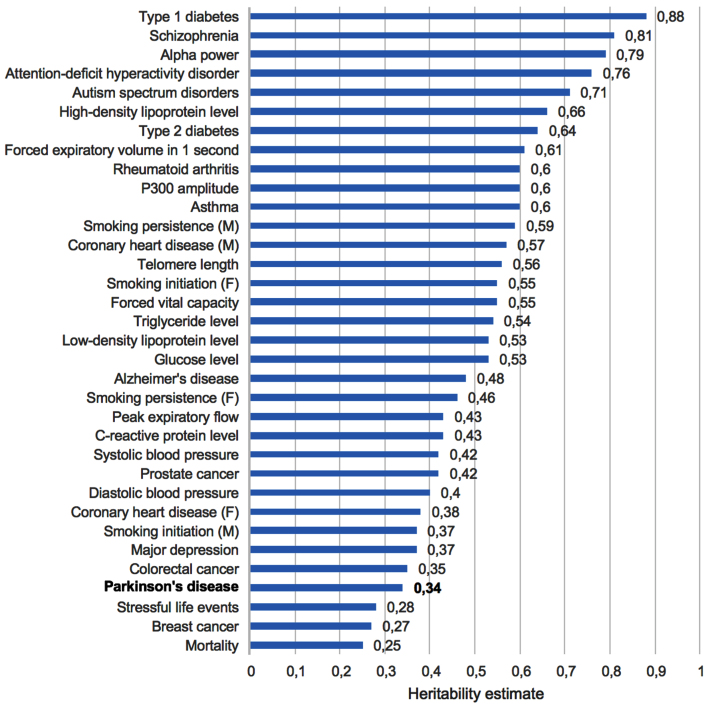
Heritability estimates from twin studies. Adapted from: [27].

Twin studies have been performed for a variety of clinical endpoints and diseases to evaluate the relative contribution of genetics to the disease etiology [27]. PD has also been studied in this regard. We have rearranged the findings of these twin studies by ranking the different diseases according to the relative magnitude of the estimated genetic contribution (Fig. 1). Interestingly, when PD (with an estimated heritability estimate of 0.34) is then considered in perspective to other common disorders with a mixed etiology, PD is almost at the bottom of the list, emphasizing the relatively modest genetic contribution to the etiology of this disease, certainly when compared to other conditions where the genetic contribution is much more substantial [27]. In a different twin study with an even longer follow-up, the concordance in monozygotic twins was 0.20, but in dizygotic twins 0.13, also indicating a substantial (shared) environmental effect [28]. This included early-onset PD, which typically has a larger genetic contribution. Large GWAS have identified up to 90 risk loci, and estimated that these variants may account for approximately 22% of PD risk, but these variants are estimated to be hundreds of thousands years old [14].

## ETIOLOGY SPECTRUM

**Fig. 2:**
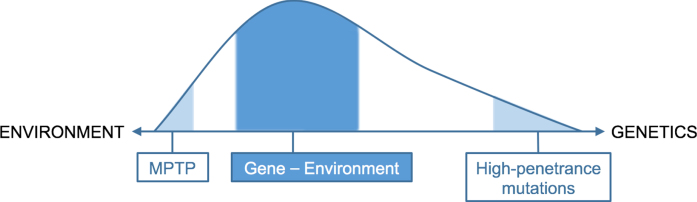
Proposed schematic distribution of etiology of PD, with a skew favoring environmental factors.

Taken together, only a rather small proportion of PD cases can be explained solely by either genetics (i.e., full-penetrance mutations) or by just the environment (e.g., exposure to the neurotoxin MPTP). For the majority of patients, the etiology of PD likely depends on an interaction between these two factors, such that a genetic predisposition modulates the personal risk when exposure to an environmental toxin occurs. Emerging pre-clinical research indeed indicates a synergistic effect between various genetic and environmental factors [29], as previously emphasized for the example of *GBA1*. These findings suggests that a person’s genotype influences his or her susceptibility to or resilience against environmental factors, and therefore represents a co-determining factor that determines the overall risk for developing PD. Considering the old age of most currently known genetic risk factors compared to the age of the PD phenotype, we depict this etiology spectrum as a skewed graph, favoring a relatively larger contribution by environmental factors (Fig. 2). Next to gene-environment interactions, other interactions are very likely to contribute to the development of PD. This includes environment-environment interactions, such as head trauma and pesticides [30] or exposure to combinations of multiple different pesticides [8], and interaction with modifiers, such as lifestyle factors [31]. Taken together, a combination of different factors, either gene-environment interaction or co-exposure to several environmental factors, is responsible for the development of PD, with different factors predominating in different people with PD. Identification of the contribution of each of these factors is therefore of great importance.

## CONCLUSION

We have presented two perspectives from the field of genetics that would appear to emphasize that environmental factors take a principal role in the etiology of PD. While elucidation of the involved genetic factors can provide essential insights into the involved pathophysiological processes, identification of responsible environmental factors (many of which appear to be man-made) is paramount from a perspective of prevention. Such prevention measures will become essential if we want to stop the rapid growth of PD worldwide.
